# Development of an Asthma Home-Visit Training Program for Community Health Workers and Their Supervisors in Washington State

**DOI:** 10.3389/fpubh.2021.674843

**Published:** 2021-06-25

**Authors:** Nuha Elkugia, Mary E. Crocker, James W. Stout, Kaylin Bolt, Bryan J. Weiner, C. Bradley Kramer

**Affiliations:** ^1^Chronic Disease and Injury Prevention Section, Public Health-Seattle and King County, Seattle, WA, United States; ^2^Division of Pulmonary and Sleep Medicine, Department of Pediatrics, University of Washington, Seattle, WA, United States; ^3^Pulmonary and Sleep Medicine, Seattle Children's Hospital, Seattle, WA, United States; ^4^Division of General Pediatrics, Department of Pediatrics, University of Washington, Seattle, WA, United States; ^5^Department of Health Services, University of Washington, Seattle, WA, United States; ^6^Assessment Policy Development and Evaluation Unit, Public Health-Seattle and King County, Seattle, WA, United States; ^7^Department of Global Health, University of Washington, Seattle, WA, United States

**Keywords:** community health worker, asthma, home visit, community based participatory research, health disparities, environmental assessment, implementation science, training

## Abstract

The community health worker (CHW) asthma home-visiting model developed by Public Health-Seattle & King County (PHSKC) is an evidence-based approach proven to improve health outcomes and quality of life. In addition, it has been shown to be an effective and culturally appropriate approach to helping people with asthma understand the environmental and behavioral causes of uncontrolled asthma, while acquiring the skills they need to control their asthma. This paper describes the development and implementation of training curricula for CHWs and supervisors in the asthma home visiting program. To facilitate dissemination, this program took advantage of the current healthcare landscape in Washington State resulting from Centers for Medicare & Medicaid Services (CMS) approval of the 1115 Medicaid Waiver project. Key aspects of the training program development included: (1) Engagement: forming a Community Advisory Board with multiple stakeholders to help prioritize training content; (2) Curriculum Development: building the training on evidence-based home-visit protocols previously developed at PHSKC; (3) Implementation of the training program; (4) Evaluation of the training; and (5) Adaptation of the training based on lessons learned. We describe key factors in the training program's improvement including the use of a community-based participatory approach to engage stakeholders at multiple phases of the project and ensure regional adaption; combining in-person and online modules for delivery; and holding learning collaboratives for post-training and technical support. We also outline our training program evaluation plan and the planned evaluation of the home visit program which the trainees will deliver, both of which follow the RE-AIM framework. However, because the COVID-19 pandemic has curtailed training activities and prohibited the trainees from implementation of these CHW home visit practices, our evaluation is currently incomplete. Therefore, this case study provides insight into the adaptation of the training program, but not the delivery of the home visit program, the outcomes of which remain to be seen.

## Introduction

Asthma is a chronic condition that impacts the lives of ~1 in 12 Americans and over 24 million people in the United States (1). The rates of asthma are highest among Hispanics and non-Hispanic Black communities who have been historically underserved and underinsured in the United States (2, 3). In Washington State, an estimated 490,000 adults and nearly 110,000 children have asthma, and mirroring the rest of the nation, the condition disproportionately impacts low-income and minority populations (4–6). The high cost of medical care for asthma, the lost school and workdays, and reduced quality of life for individuals living with the disease make asthma a major health priority (7).

Many of the drivers of asthma morbidity are factors related to low socioeconomic status, such as transportation, language, and financial barriers; poor access to primary health care services; and environmental exposures to allergens and irritants including second-hand smoke (3, 8–13). Many of these factors can be addressed by interventions conducted by community health workers (CHWs). Asthma CHWs are typically lay health workers who have been trained by medical or other health providers to deliver health education regarding asthma. They come from the communities in which they serve and have a familiarity with asthma and its impact on patients and families. Home visits bring CHWs into the settings where environmental exposures are active, allowing them to address asthma triggers and more directly promote health behavior change. To reinforce the behavior change, the CHW models trigger reduction strategies and provides the clients with supplies, such as mold cleaning kits, mattress encasements to reduce exposure to dust mites, and vacuums with high-efficiency particulate air (HEPA) filters. CHWs can also deliver education on medication use and self-management of asthma while connecting families to resources to overcome barriers to symptom management. In the best scenarios, CHWs then connect with the patient's primary care physician and their health plan to inform a longer-term care plan that maintains support for both home environment and medication adherence.

These multicomponent home visit interventions for asthma led by CHWs have been shown to reduce morbidity from asthma and result in cost savings to the overall health care system (14–16). Multicomponent home visits for asthma have also been recommended by the Task Force on Community Preventive Services to reduce asthma morbidity (17). However, a lack of consistent state or federal funding for these efforts has impeded a widespread scale-up of the model. In Washington State, the Medicaid waiver project, fueled by a $1.5 billion 1115 Medicaid Waiver, has created an opportunity for the scale-up of an evidence-based asthma home visit intervention through delivery of a training program for CHWs and CHW supervisors. This paper describes the CHW training program by outlining the five phases of our process: engagement, curriculum development, implementation, evaluation of the training, and adaptation. We then discuss challenges and lessons learned.

## Context

Public Health-Seattle & King County (PHSKC), the health department of the most populous county in Washington State, has prioritized reduction of asthma health disparities using a CHW-led intervention model. The CHW program at PHSKC has many years of experience in development and implementation of CHW-led public health programs for asthma, including five research studies spanning from 2004 to 2015 (16, 18–21). Building on this experience, PHSKC, in collaboration with the University of Washington, conducted the Guidelines to Practice (G2P) study funded by the Patient-Centered Outcomes Research Institute (22). This randomized controlled trial conducted from 2015 to 2017 examined the effectiveness of CHW home visits including home environmental assessment for identification of asthma triggers, education on self-management plans and medication use. The trial also highlighted the benefits of collaboration between the CHWs and the patient's primary care provider and Medicaid managed care plan. Results from the G2P study showed that individuals who received home visits from a CHW experienced more days without asthma symptoms, fewer nights when they woke up because of asthma, and fewer missed work or school days compared to those who did not receive home visits (22).

This success motivated us to scale up the CHW asthma home visit model used by PHSKC to other communities throughout Washington State. Historically, there has been no sustainable funding source for such a project. However, the recent initiation of the $1.5 billion 1115 Medicaid Waiver project in Washington created an opportunity (23). The Waiver project is administrated through the Centers for Medicare & Medicaid Services (CMS) and allows states to waive certain federal funding rules and commit to demonstration projects which are cost-neutral or money-saving to the health care system, provided states reach certain health metrics. In Washington, the Waiver projects are chosen and implemented by regional organizations called Accountable Communities of Health (ACHs). For the current cycle of projects, several of the nine ACHs in Washington selected community-based asthma management as a strategy to focus on chronic disease prevention and control. Our evidence-based CHW asthma home visiting program fit several ACH communities' goals, which opened the door for a collaboration ultimately including several ACHs.

The project described here is hosted by PHSKC with four primary implementation partners within western Washington: three ACHs (King County, Cascade-Pacific, and Southwest Washington) and one Federally Qualified Health Center (FQHC, SeaMar), a safety-net provider with clinics in nine counties in Western Washington (Table 1). These partners each serve as hubs or central organizing bodies for multiple clinic- and community-based organizations that employ CHWs to provide chronic disease and health behavior change programs.

**Table 1:** Community characteristics.

**Region**	**Estimated CHW FTE**	**Medicaid population**	**Medicaid asthma population**	**Estimated population with poorly controlled asthma**	**Estimated eligible and interested in home-visit program**
HealthierHere: King county accountable community of health	34.0	432,633	26,598	3,300	1,650
Cascade-pacific action alliance	14.0	189,442	11,000	1,400	700
Southwest washington accountable community of health (SWATCH)	10.0	135,091	8,000	1,000	500
SeaMar Community health centers	7.0	206,698	12,951	1,600	800

*CHW, community health worker; FTE, full-time effort*.

We sought to train the CHWs connected to these regional hubs on the delivery of the evidence-based asthma home visiting program. However, we realized that our CHW program had 25 years of development and infrastructure that may contribute to positive results not easily replicable by other programs. In order to boost regional program effectiveness and sustainability, we decided to develop a program manager/supervisor training as well. This training would be built on the principles of best practices in creating supportive and sustainable CHW programs (24–26).

The development and implementation of our training program followed five phases: (1) Engagement: we establish our community advisory board, partnership, and infrastructure for decision-making to support throughout the project. (2) Curriculum Development: we develop our training curriculum. (3) Implementation: we host our first trainings. (4) Evaluation: we measure and assess how the trainings were received. (5) Adaptation: we iterate phases 2, 3, and 4 applying improvements based on learnings from the implementation and evaluation stages. This manuscript is intended to present the proposal, design, and development of a training program, following many years of research and development of the program. At the time of submitting this manuscript, we are between phases 4 and 5. Therefore, we present some initial (i.e., preliminary) outcomes and our plan for the Adaptation phase. This work was funded by the Patient-Centered Outcomes Research Institute (PCORI).

## Key Programmatic Elements

### Phase 1: Engagement

Our work approaches community, program development, and research based on the principals of community based participatory research (CBPR) (27). Using this approach, program and evaluation plans are generated by all stakeholders involved, including patients, clinicians, community groups, payors, academics, and researchers. Through the application of CBPR principles to our work, we ensure that resources, decision-making power, and ideas are collectively shared.

Our initial step was to convene a Community Advisory Board (CAB) consisting of key stakeholders who would provide guidance and increase local engagement. The CAB included representatives from our priority patient populations, CHW and supervisor teams, Washington State's Department of Health CHW program, local managed care organizations and Medicaid, a Federally Qualified Health Center, PHSKC, and three additional regional ACHs. Three CHWs from PHSKC served as lead educational consultants, and a University of Washington research group specializing in producing online, interactive medical training resources provided expertise. The CAB was representative of both new stakeholders as well as partners who were involved in our recent PCORI-funded randomized trial. This was intentional so that the diversity of experiences and expertise from our partners would enable adaptation within local contexts and provide a feedback loop regarding implementation challenges and opportunities. Authentic engagement, clear communication, and shared leadership with patients and other stakeholders in all project phases was considered critical to the project's success.

We held regular monthly CAB meetings during which we created working groups for different tasks, and encouraged active participation toward four primary goals: (1) improve the scope and design of the training program to meet the needs of the various project partners; (2) develop, tailor, and deliver the support structures needed by the CHWs, program supervisors and organizations implementing the model; (3) provide input on the design of the evaluation of the training program, and (4) engage stakeholder end-users and funders to determine opportunities for continued spread. Participation in CAB meetings ranged from 20 to 30 in attendance, beginning in March 2020 and continuing to the present.

Participation in the CAB was compensated with an annual stipend offered to our four project partner agencies through funds from our PCORI grant. The amount was calculated based on the anticipated staff time and resources, and use of the stipend was not restricted. In addition, we developed several additional entry points for engagement that were fully compensated. Each site was encouraged to identify at least one CHW, one program manager, and one person with asthma from their region to sit on the CAB. We also budgeted for interviewing program recipients in each region to provide feedback on the program aspects they experienced, compensating them for their time.

### Phase 2: Curriculum Development

After assembling the CAB, we conducted an in-depth examination of the existing evidence-based CHW asthma educational and environmental protocols at PHSKC and resources on CHW supervision to create a comprehensive foundation on which to build the new curriculum. We strategized with the CAB to narrow down the protocols to those most relevant and useful, and to identify gaps in information. The resulting protocols were then modified into a format appropriate for an in-person 2-day training for CHWs. Selected content was converted into a multi-media interactive online tutorial for asthma-focused CHWs by our partners at the University of Washington.

In order to assist the integration of CHWs into their teams and to increase the sustainability of the project, we also designed a training specifically for CHW supervisors. We used the same strategy described above to develop a curriculum for a 2-day training for CHW supervisors.

During the monthly meetings of the CAB, we worked together on the above curriculum development tasks and solicited feedback on the materials developed. The CAB provided invaluable feedback regarding potential implementation challenges and opportunities for continued dissemination of this model. For example, one of our primary partners noted that their CHWs primarily work with asthma patients who also are experiencing homelessness. Based on that feedback we incorporated information in our trainings on how CHWs can be innovative when it comes to delivering the asthma intervention protocols in situations where traditional housing or indoor shelter is not an option.

### Phase 3: Implementation

The application of a community-based collaborative approach paired with an adult learner-centered curriculum development process resulted in a training tailored to support CHWs focused on asthma and their supervisors. This process yielded a collection of online tutorials intended for independent, self-paced study (available at chw.uwimtr.org), to be followed by an in-person training, covering the topics listed in Table 2. The combination of online and in-person study was intended to reach participants of different learning styles and to engage non-native English speakers who preferred an in-person interactive training where interpretation was provided. By holding multiple trainings over the project years, our goal was to have some CHWs or program managers step forward in each region to serve as a regional Asthma Champion that can help observe and provide peer-support and make connections for future training.

**Table 2:** Training components.

**Training format**	**Module topic**	**Content**
Online tutorial for CHWs	Asthma: the basics	• Asthma definition • Triggers and allergens • Definition of asthma control • Controller vs. rescue medication • Importance of flu shots • CHW assessment, key messages, and actions
	Medication adherence and guidelines for using an asthma action plan	• Strategies to improve medication adherence • Asthma action plan • How to use an inhaler • When to use a spacer
	Respiratory distress: warning signs and responses	• Symptoms and warning signs in the individual patient • Warning signs and the asthma action plan • What to do during an acute asthma episode • When immediate medical care is necessary • Correlation between colds and asthma
	Home environmental check and cleaning techniques	• How to complete a Home Environmental Checklist • Contents of Safer Cleaning Kit • How to assess for cleaning issues • How to clean various rooms
	Dust control guidelines: vacuuming and doormats	• Importance of reducing dust exposure • Importance of using a door mat • How to vacuum • How to maintain a vacuum cleaner
	Dust mite guidelines	• Importance of reducing dust mite exposure • How to control dust mites
	Mold and moisture guidelines	• Common sources of moisture in the home • Importance of vapor barriers • How to clean mold • How cold homes affect people with asthma
	Roach and rodent guidelines	• What is integrated pest management, and why is it important • The cockroach elimination process • Cockroach management strategies • Rodent control strategies • Prevention of rodent problems
	Pets: management and HEPA filter use	• Which pets can be asthma triggers • Actions to reduce pets' effects on asthma • HEPA filters and how to clean them
	Environmental air exposure: indoor and outdoor	• How tobacco smoke affects asthmatics • Why children are more susceptible to smoke • How to reduce pollen exposure • How to be prepared for wildfire smoke
In-person CHW training	Trauma-informed care	• Definitions of trauma and trauma informed care • Adverse Childhood Experiences (ACEs) study and the ACEs quiz • Limitations of the ACEs studies • CHWs as frontline workers
	Self-care for CHWs	• Boundaries between CHWs and patients • Prioritizing self-care • Stress, secondary trauma, and resilience
	Motivational interviewing	• Working with chronic disease patients • How to ask open-ended questions • Engaging in reflective listening • How to use motivational interviewing with asthma patients
	Asthma medication tools	• Types of asthma medications • How to explain medications to patients • Practice with placebo tools • Challenges when working with patients using their medications
	Case studies and role play	• Small group review of case studies utilizing variety of protocols • Large group discussion of strategies used and concerns raised
In-person CHW supervisor training	CHWs' uniqueness	Understanding their roles, skills, strengths, and challenges
	Best practices for supervising CHWs	Individual vs. group supervision and shadowing
	Integration	Effectively integrating CHWs into organizations
	Building bridges	Building bridges between CHWs, members of organizations, and external partners

#### Online Tutorials

The online tutorials covered the topics listed in Table 1, and take an estimated 2–3 hours to complete. The modules included interactive elements such as video demonstrations by CHWs, quiz questions, and visually rich graphics tailored to educate and engage participants. For example, a detailed picture showing differences between a normal lung and a lung affected by asthma were intended to help CHWs describe asthma and its symptoms. The CHWs registered for trainings were encouraged to complete these modules prior to attending the in-person training. Those completing the modules were presented certificates of completion at the in-person event.

#### In-person CHW Training

The in-person training curriculum topics (Table 1) were implemented by our support staff, CHW education specialists from PHSKC, a pediatrician specializing in asthma, and a motivational interviewing trainer. Training took place over two full days, with a total of 6.5 hours of instruction each day. Interpretation in Spanish was provided for non-English speaking CHWs. During the sessions, the PHSKC CHWs modeled how to use the protocols and conduct an asthma home visit, and the trainees were given the opportunity to practice with their peers during case studies and role play sessions.

A key component of the in-person training was the distribution of supplies which were intended for the CHWs to reuse for future trainings. We provided an environmental and educational protocol handbook, patient asthma education packets, safer cleaning kits with healthy cleaning methods, and placebo medication tools that allowed demonstrations of medication technique. The training and supplies were offered free of charge, funded by our grant.

#### In-person Supervisor Training

The CHW supervisor has an important role in the success of any CHW program. We hosted a request for proposals to solicit a curriculum developer with experience with a broad set of CHW programs nationally. This contractor focused on the specific elements that made the existing asthma home visit program at PHSKC most successful. The topics covered are detailed in Table 1. The training first focused on defining the role of CHWs in order to foster support, mentoring, and supervision. Elements of 360 supervision discussed included group supervision, 1-on-1 supportive supervision, direct observation, and patient feedback (28). The training also highlighted methods for promoting relationships to support CHWs and building multi-sector relationships with clinical and community efforts. In addition, the training discusses continuous professional development for the CHW, establishing formal connections into the health care system, supporting community outreach, and compensation and workload management (24). Finally, the backbone of our CHW program is rooted in a philosophy of trauma-informed principles (29, 30), motivational interviewing, and self-care.

#### Monthly Learning Collaboratives and Ongoing Training Support

After implementing the trainings, we held monthly virtual meetings termed Learning Collaboratives for all past trainees in order to provide technical assistance and one-to-one support, create an environment for cross-learning among CHW peers, and provide ride-along or shadowing opportunities as requested. Other specific agenda topics have been largely driven by attendees. These Learning Collaboratives were also useful during our evaluation phase as a setting in which to conduct focus groups and solicit feedback on evaluation results. These meetings are intended to continue beyond the adaptation phase.

### Phase 4: Evaluation

Our evaluation plan focuses on the impact of the training program, but eventually will also assess the public health impact of the home-visit program that is delivered by the trainees (Table 3). This evaluation plan was developed with the guidance of an implementation science expert and applies the RE-AIM framework, which provides a practical approach for evaluating programs within “real world” settings in the domains of Reach, Effectiveness, Adoption, Implementation, and Maintenance (31). The RE-AIM framework balances internal and external validity and addresses considerations relevant to dissemination, implementation, and scale-up. It is also compatible with socio-ecological models of health and useful for evaluating the public health impact of multilevel, multicomponent programs such as the PHSKC asthma home visit intervention.

**Table 3:** Evaluation plan.

**RE-AIM metric**	**Training program**	**Home visit program**
Reach	• Number, proportion, and characteristics of CHWs and supervisors who attended trainings	• Number, proportion, and representativeness of Medicaid patients (ages 5–65) in each ACH catchment area with severe, uncontrolled asthma visited by a CHW
Effectiveness	• Change in trainee confidence, comfort, and knowledge; ability to apply new skills and knowledge; and trainee opinion on clarity of information	• Patient-reported symptom-free days • Hospitalization and ED use • Rescue medication use • Missed days of work or school • Self-rated asthma control over the past 4 weeks
Adoption	• Components of content which were/were not used by trainees • Number and proportion of ACH member organizations that support CHWs attending the training program and making asthma-related home visits	• Number and proportion of CHWs who make their first home visit
Implementation	• Number of trainings conducted • Perceived facilitators, barriers, and adaptations of trainees when applying the skills or knowledge learned	• Program fidelity • Number of home visits • Number of new referral pathways established during the program • Facilitators, barriers, and adaptations to program delivery
Maintenance	• Number and proportion of ACH member organizations that support CHWs attending the training program and making asthma-related home visits at 6 months (or longer) post-training • Number of partners, resources, or referral pathways for asthma related services developed by an organization	• Number and proportion of CHWs who make their first home visit at 6 months (or longer) post-training

The evaluation of the training program takes a mixed-methods approach including review of program records, surveys of all trainee participants, and interviews of a subset of trainees. Program records were reviewed to elucidate process measures such as numbers of attendees and trainings delivered. Pre-post surveys were administered to CHW and supervisors to assess change in confidence, comfort and knowledge, ability to apply new skills and knowledge, and opinion on clarity of information. Finally, we also conducted interviews with a subset of CHWs and CHW supervisors who attended trainings. These interviews covered barriers to client behavior change, experience at the trainings, implementation of knowledge/tools gained, the impact of COVID-19 on their work, and desired future trainings.

### Phase 5: Adaptation

Our initial qualitative interviews and survey data from the trainees will be utilized to iteratively evaluate our trainings and inform program improvements. The implementation team will compile results and present suggested next steps for revisions to the CAB for advice and approval. With their guidance, the implementation team will adapt, schedule, and deliver the revised trainings to each of the partner sites. New trainees will provide feedback for review allowing one final opportunity for revision.

## Preliminary Results

Our training program commenced in January 2020. Shortly thereafter, the COVID-19 pandemic took hold in Washington State, which rapidly led to restrictions on in-person gatherings and shifting of heath department priorities toward the pandemic rather than chronic disease. Both in-person trainings and asthma home visits were suspended, which prevented new trainees from returning to their communities to apply what they had just learned. As a result, currently we have only partial results from our evaluation of the training program (presented below), but no data regarding the home visit intervention. The evaluation will continue when in-person asthma CHW activities are allowed to resume.

In January and February of 2020, we delivered two in-person CHW trainings and two in-person CHW supervisor trainings, rather than the planned four trainings for each group (one for each ACH region). Our initial plan to hold separate trainings in each ACH region was modified once we discovered established venues that were convenient to multiple regions, allowing us to consolidate to two locations. A total of 60 individuals attended a training. This initial cohort of trainees were representative of over 12 different organizations including health departments, local neighborhood groups, community-based organizations, and managed care organizations. The training was provided in Spanish and English as planned.

Initial results from surveys of training participants showed the following:

- 77% of CHWs (24 out of 31) were extremely clear about the takeaway points.
- 91% of supervisors (32 out of 35) were extremely clear about the takeaway points.
- 74% of CHWs (23 out of 31) thought the presentation of information was extremely clear.
- 89% of supervisors (32 out of 36) thought the presentation of information was extremely clear.
- 71% of CHWs (22 out of 31) were rated their confidence 8 or higher for their ability to apply what they learned at work (scale of 0 = not at all and 10 = extremely confident).
- 91% of supervisors (31 our 34) rated their confidence 8 or higher for their ability to apply what they learned at work (scale of 0 = not at all and 10 = extremely confident)

Interviews revealed that overall, there was a positive experience with major elements of the training such as food, environment, location, built confidence, facilitation, activities, interactivity and engagement, and room set-up and rotation. When speaking about the facilitation, one participant stated, “I think any time you have a training that's gonna be 16 hours over two days and there's a lot of material to cover, the facilitation makes or breaks the training. And they were just so personable and open, and asked pertinent questions and waited for the replies that needed to come out. I didn't feel pressured in any way to perform or not perform. I think the facilitators make or break any training, and they were exceptional.”

CHWs especially felt that the motivational interviewing, visual aids/diagrams, role playing practice and the background was helpful. One CHW stated, “What will help me are the handouts that they gave us on ‘What is asthma?' and ‘How do we breathe?' [and] on why it's important to have controlled asthma and the importance of what the inhalers do. The actual visual diagrams I think will help clients understand ‘Oh yes, this is important', since they'll be able to see how it affects them when they are having an asthma attack, what actually is happening inside their lungs.”

Several areas for improvement of the training program were identified. For example, feedback from the CHW training showed the desire for a refresher course online, funding for supplies, instruction on topics beyond asthma, and avoiding repetition of the online module content. Supervisors suggested slowing the pace of the training, providing tools that can also be applied to other chronic conditions, and spending more time on best practices for supportive supervision. One supervisor also suggested including a half-day overlap with CHWs to share learnings.

## Discussion and Limitations

This report has outlined the development of a training program to teach CHWs and CHW supervisors how to deliver an asthma home visit intervention built on an evidence-based, cost-effective model in Washington State. The scale-up of this approach took advantage of the state Medicaid Waiver program, which is likely to accelerate its integration into the health care system and improve its sustainability over time. Our use of a Community Advisory Board from the very early stages of the project enabled us to identify opportunities for adaptation of the proposed training to the settings in which it would be delivered, and offered invaluable feedback on what was and was not working. Following the trainings, the monthly Learning Collaboratives allowed ongoing engagement and review of skills, which is also expected to improve effectiveness and sustainability. The iterative nature of the curriculum design allows for continual improvement of the program over time. While not all states are participants in a Medicaid Waiver project, the other community-based participatory methods described here may still be applicable for other organizations seeking to scale up CHW home visit models across different settings in their state.

Throughout the implementation of this project, we learned several lessons which will help to improve future iterations of the program. Firstly, changing conditions during the pandemic reinforced the importance of flexibility and willingness to adapt program implementation to the setting. Since the COVID-19 pandemic started in our region very shortly after our in-person trainings, many trainees were unable to go back to their communities in person and immediately apply their new asthma education skills. As the pandemic continues and some attention shifts back toward non-COVID-19 health concerns, we are encouraging our trainees to begin conducting virtual asthma visits as a way to resume providing this service. We are also developing ways for trainees to refresh these asthma skills remotely. Another invaluable lesson was the tremendous utility of soliciting feedback from both the community and CHW participants early on and frequently. This provided the insight we needed into the needs of each individual community, such as a focus on managing asthma during housing instability, or the importance of obtaining funding for program supplies. Recognizing the challenging and complex environments the trainees were working in was essential to adapting the training appropriately.

Our program has a number of limitations which are important to consider. Firstly, the training capitalized on the Washington Medicaid Waiver project. While any state can apply for permission to pursue similar projects through the Centers for Medicare and Medicaid Services, they do not currently exist in every state, which limits the generalizability of our program design. Additionally, our program is funded by an outside source (PCORI). In accordance with PCORI values, we have done our best to avoid direct compensation for program aspects that would require coverage by an employer or the health system to be sustainable. However, we have offered compensation for the CAB and the training program staff, and the training and materials are offered free of charge, which raises concerns for sustainability. The Learning Collaboratives are also dependent on this funding as it provides for program staff. Because of this, our team has been exploring avenues of more sustainable funding, including the potential for Medicaid reimbursement of material costs and CHW time, but this issue has not yet been resolved.

Despite these limitations, our case study has shown that a community-based approach to curriculum development and improvement can produce a training program that is adaptable, engaging, and valuable to participants and has the potential to produce CHWs competent to deliver high-quality, effective home visit interventions for asthma. The coronavirus pandemic has limited the ability of our trainees to implement their training, but our program is adapting with the creation of protocols for virtual visits. The next steps include evaluation of the effectiveness of our trainees' services, and further refinement of the training program design. Through this community-based, adaptive approach, we hope to harness the proven effectiveness of CHW-led home visits to address asthma in a way that is culturally acceptable and cost-effective, and addresses some of the root causes of asthma-related disparities.

## Data Availability Statement

The raw data supporting the conclusions of this article will be made available by the authors, without undue reservation.

## Ethics Statement

The studies involving human participants were reviewed and approved by University of Washington Institutional Review Board. Written informed consent for participation was not required for this study in accordance with the national legislation and the institutional requirements.

## Author Contributions

JS, BW, and CK contributed to the conception and design of the program and evaluation plan. NE, JS, KB, and CK implemented the program. NE wrote the first draft of the manuscript. NE, MC, KB, and CK wrote sections of the manuscript. All authors contributed to the article and approved the submitted version.

## Conflict of Interest

The authors declare that the research was conducted in the absence of any commercial or financial relationships that could be construed as a potential conflict of interest.
